# Robotic Versus Hand-Assisted Distal Pancreatectomy: A Comparative Single Center Retrospective Study

**DOI:** 10.3390/jcm14144919

**Published:** 2025-07-11

**Authors:** Nabih Essami, Esther Kazlow, Eitan Dines, Aasem Abu Shtaya, Wisam Assaf, Riad Haddad, Ahmad Mahamid

**Affiliations:** 1 Technion Israel Institute of Technology, Rappaport Faculty of Medicine, Haifa 3109601, Israel; 2 Carmel Medical Center, Department of Surgery, Haifa 3436212, Israel; 3 Carmel Medical Center, Department of Gastroenterology, Haifa 3436212, Israel; 4 Carmel Medical Center, Department of Obstetrics and Gynecology, Haifa 3436212, Israel

**Keywords:** pancreatectomy, robotic surgery, robotic-assisted distal pancreatectomy, hand-assisted laparoscopic surgery, postoperative outcomes

## Abstract

**Background**: While there is an abundance of comparative studies on open, laparoscopic, and robotic-assisted distal pancreatectomies (RDPs) available in the literature, direct comparisons between RDP and hand-assisted laparoscopic distal pancreatectomy (HALDP) are limited. This study aimed to assess the safety and efficacy of RDPs in comparison to HALDPs in the treatment of pancreatic lesions. **Methods**: This study reviewed 97 patients who underwent distal pancreatectomy at Carmel Medical Center between 2008 and 2024. After excluding 40 patients (24 open and 16 pure laparoscopic resections), the final cohort comprised 57 patients: 20 RDPs and 37 HALDPs. The primary outcomes included peri-operative parameters, while secondary outcomes encompassed 90-day morbidity and mortality. **Results**: RDPs led to significantly longer operative times (3.9 vs. 2.5 h, *p* < 0.001) but resulted in shorter hospital stays (4.7 vs. 5.8 days, *p* = 0.02) and a higher number of harvested lymph nodes (11 vs. 5.4, *p* = 0.01). While clinically significant pancreatic fistula rates were numerically higher in the RDP group (35% vs. 16.2%, *p* = 0.18), this difference was not statistically significant. Overall, complication rates were comparable (55% vs. 43.2%, *p* = 0.39). Severe morbidity (Clavien–Dindo ≥ IIIa) was absent in the RDP group compared to 8% in the HALDP group (*p* = 0.04). No 90-day mortality was observed in either group. **Conclusions**: This study indicates that although RDP involves longer operative times, it may provide certain advantages for patients, such as shorter hospital stays, better lymph node retrieval, and a notable decrease in postoperative morbidity when compared to HALDP. Larger prospective studies are needed to validate these results and to determine the most effective surgical approach for distal pancreatectomy.

## 1. Introduction

Pancreatic cancer is an aggressive malignancy with a poor prognosis, frequently diagnosed at an advanced stage due to the absence of early symptoms and effective screening methods. It predominantly affects older adults and is marked by rapid tumor growth and early metastasis. Despite advancements in surgical techniques, chemotherapy, and radiation therapy, pancreatic cancer continues to be one of the leading causes of cancer-related deaths worldwide [1]. Surgical resection remains the only potentially curative treatment option for patients with localized disease [2]. Distal pancreatectomy, a procedure performed to remove tumors in the body and tail of the pancreas, is indicated for various pancreatic lesions, including benign, premalignant, and malignant conditions [3,4].

The optimal surgical approach for distal pancreatectomy depends on a combination of anatomical characteristics, patient-specific factors, and surgical expertise. Traditional open surgery provides direct visualization and access, which is especially advantageous for patients with large tumors or vascular involvement. In contrast, laparoscopic techniques typically lead to shorter hospital stays, less postoperative pain, and reduced blood loss while achieving oncologic outcomes similar to those seen with open surgery [5]. Hand-assisted laparoscopic surgery (HALS) combines the benefits of both open and laparoscopic techniques, providing surgeons with useful tactile feedback while maintaining the advantages of minimally invasive procedures [6]. Compared to open surgery, HALS has been shown to result in shorter operative times, reduced blood loss, and shorter hospital stays, all while maintaining comparable oncologic outcomes [6]. When compared to traditional laparoscopic approaches, HALS exhibits lower conversion rates to open surgery and shorter operative times while achieving similar complication rates, including for postoperative clinically relevant pancreatic fistulas [7].

Robot-assisted distal pancreatectomy (RDP) enhances surgical precision through the use of robotic arms, a surgeon’s console, and 3D visualization [8]. Recent studies and meta-analyses comparing RDP to traditional laparoscopic procedures suggest that both offer comparable outcomes in terms of operative time, complications, blood loss, transfusion rates, and other key metrics [9,10,11,12,13]. However, RDP has shown potential advantages, such as a lower conversion rate, better spleen preservation, a higher number of harvested lymph nodes [11], and potentially better oncological outcomes in pancreatic ductal adenocarcinoma with a higher R0 resection rate [12]. Additionally, RDP may be associated with a shorter hospital stay compared to the purely laparoscopic approach [13].

Although extensive research has compared various surgical techniques for distal pancreatectomy, there remains a significant gap in direct comparisons between robotic and hand-assisted approaches. Robotic surgery is known for its superior visualization and ergonomics, while HALS offers essential tactile feedback during intricate dissections. Additionally, in contrast to robotic surgery, HALS is cost-effective and has a shorter learning curve, which is beneficial for medical centers that are transitioning to minimally invasive methods.

While comprehensive comparisons involving open and pure laparoscopic techniques are well-documented in the literature, direct comparisons between the robotic and hand-assisted platforms are lacking. These two advanced minimally invasive approaches offer different advantages, with robotic surgery providing superior ergonomics and visualization while HALS preserves crucial tactile feedback. Given this, our study was specifically designed to isolate and compare these two techniques to provide focused evidence for centers navigating the choice between them. Therefore, to ensure a targeted and homogenous comparison, patients who underwent open or pure laparoscopic resections during the study period were excluded from this analysis.

Our study aims to evaluate the safety and efficacy of RDP versus HALDP as selecting the optimal approach can improve patient outcomes, reduce costs, and increase procedural efficiency. Understanding the strengths and limitations of each technique will allow surgeons to make more informed decisions and deliver better patient care.

## 2. Materials and Methods

This study retrospectively identified all patients who underwent a distal pancreatectomy at Carmel Medical Center (Haifa, Israel) between January 2008 and December 2024. Our institution is a university-affiliated, tertiary-care hospital featuring a high-volume hepato-biliary unit with more than two decades of expertise in advanced hepato-pancreato-biliary surgery. All patient data, including detailed demographic and perioperative information, were retrieved from our comprehensive electronic medical record system, known as the Chameleon database. The research protocol was approved by the Institutional Review Board (IRB) of Carmel Medical Center and was performed in adherence with the ethical principles of the Declaration of Helsinki and Good Clinical Practice Guidelines. The data review process involved a thorough examination of patient demographics, comprehensive surgical histories, final pathology reports, and all follow-up notes from the medical and oncology teams. Prior to their procedures, every patient obtained clearance from the anesthesiology department and was managed through a multidisciplinary team approach. For the surgical procedure itself, all patients provided written informed consent after a detailed discussion of the potential risks and benefits. However, due to the retrospective design of this research, the Institutional Review Board Committee waived the need for separate patient consent for study participation.

### 2.1. Study Population

Patients aged 18–90 years who underwent a distal pancreatectomy with robot- or hand-assisted approaches were included in this study. Patients with local unresectable disease or intra-abdominal metastasis were excluded. During the study period, 97 patients underwent a distal pancreatectomy. A total of 40 patients were excluded (24 had an open resection and 16 had a purely laparoscopic resection). The remaining 57 patients became the cohort for this study. It is important to note that due to the timeline of technology adoption at our institution, this study compares a contemporary robotic cohort (2023–2024) with a historical hand-assisted laparoscopic control group (2008–2022).

### 2.2. Surgical Technique

To ensure procedural consistency, all surgical interventions in this series were carried out by a dedicated two-person surgical team (A.M. and R.H.). Following the 2023 implementation of the Da Vinci XI surgical system within our hepato-pancreato-biliary surgery program, a transition was made to perform all subsequent distal pancreatectomies using this robotic platform. A uniform surgical methodology was applied to every patient, which involved a distal pancreatectomy combined with an en bloc splenectomy and a formal lymphadenectomy, adhering to the principles of the radical antegrade approach [14]. Management of the pancreatic remnant was also highly standardized. Specifically, transection of the pancreas was uniformly accomplished with a linear stapler, and our technique deliberately omits separate ligation or suturing of the main pancreatic duct. To complete the procedure, the cut-edge of the pancreatic stump was meticulously covered by the application of a biodegradable sealant, ensuring that it overlapped with the margins to reinforce the staple line. The level of amylase in a sample of fluid taken from the drain was measured and recorded starting from postoperative day 3.

For the robotic procedures, the Da Vinci XI surgical platform was employed. Patients were positioned supinely with their legs split, while the operating table was adjusted to a 15° right tilt and a 30° reverse Trendelenburg. An initial laparoscopic exploration of the abdominal cavity was performed prior to robotic docking. Subsequently, port placement consisted of four 8 mm robotic trocars inserted in a horizontal orientation at the umbilical level, each spaced 8 cm apart. For surgical assistance, an additional 12 mm AirSeal trocar was established at the Pfannenstiel incision point. At the conclusion of the resection, this 12 mm port was removed, and its incision, which could be extended as necessary, was used for specimen retrieval in a surgical bag. No other ports were utilized. Following the deflation of the pneumoperitoneum, all wounds were closed in layers [15]. The resected specimens were then immediately transported for pathological analysis to assess surgical margins.

For the hand-assisted laparoscopic procedures, patient positioning was identical, involving a supine, split-leg orientation with a 15° right tilt and 30° reverse Trendelenburg. The port configuration began with three trocars placed in a horizontal line at the umbilical level. An open approach was used to insert the initial 12 mm trocar just superior to the umbilicus for camera introduction. Under direct vision, two additional trocars were placed: a 12 mm AirSeal port at the left midclavicular line for assistance, and a 5 mm port at the left anterior axillary line. An incision of 7–8 cm was made in the right upper abdomen to accommodate the placement of a hand-assisted device (GelPort, Applied Medical, Rancho Santa Margarita, CA, USA). This hand port site was later used for specimen extraction. After the pneumoperitoneum was deflated, all wounds were closed in layers. Finally, the resected specimens were promptly sent for pathological evaluation of the surgical margins.

All distal pancreatectomies were performed during dedicated, fixed operating days for major HPB surgery. This institutional scheduling protocol was consistent throughout the entire study period and was not altered by the introduction of the robotic system.

Postoperative care was guided by a standardized Enhanced Recovery After Surgery (ERAS) protocol, which was a standard component of management for both cohorts. Discharge criteria were consistent for both groups, requiring patients to tolerate a soft diet, have adequate pain control with oral analgesics, be independently mobile, and have no unresolved complications, such as a clinically relevant pancreatic fistula, requiring ongoing inpatient care.

### 2.3. Outcomes

The primary analysis compared the perioperative and short-term outcomes between the RDP and HALDP groups. We assessed several variables with the potential to affect outcomes, encompassing patient demographics along with operative and postoperative data points.

To ensure consistent measurement, specific definitions were used for key metrics. Intraoperative blood loss was quantified by measuring the volume of blood suctioned from the abdominal cavity. The duration of the operation was calculated as the total time from the initial skin incision to the completion of skin closure. The postoperative length of stay represented the total number of days a patient was hospitalized, from the day of surgery to the day of discharge. Any deviation from the expected postoperative recovery path within 90 days of the procedure was classified as a complication, with severity graded according to the Clavien–Dindo classification system [16]. A postoperative pancreatic fistula (POPF) was defined and graded based on the 2016 International Study Group on Pancreatic Fistulae (ISGPF) criteria, which distinguishes between biochemical fistulae (Grade A) and clinically significant fistulae (Grades B and C) [17]. The final pathology report from the permanent specimen sections was used to determine the total number of harvested lymph nodes. A microscopic examination showing no tumor cells at the pancreas margin was defined as an R0 resection. Following their discharge from the hospital, all patients entered a follow-up protocol managed by our multidisciplinary team.

### 2.4. Statistical Analysis

The continuous variables were presented as the mean ± standard deviation or as the median with the interquartile range (IQR), depending on their distribution. Categorical variables were expressed as percentages. A comparison of demographic and clinical characteristics between the groups was performed using the chi-square test for categorical variables and the independent *t*-test or Mann–Whitney test, as appropriate, for continuing variables. A *p*-value of <0.05 was considered statistically significant. All statistical analyses were conducted using International Business Machines Corporation’s (IBM, New York, NY, USA) SPSS Statistics, version 28.

## 3. Results

This study evaluated a total of 57 patients undergoing a distal pancreatectomy, of whom 20 underwent a robot-assisted distal pancreatectomy (RDP) and 37 underwent a hand-assisted laparoscopic distal pancreatectomy (HALDP). The demographic and preoperative characteristics of the patients are summarized in Table 1. The mean age of the cohort was 64.1 ± 13.7 years, with no statistically significant difference observed between the RDP (65.2 ± 9.9) and HALDP (63.5 ± 15.5) groups (*p* = 0.65). The sex distribution was balanced across both groups, with 47.4% male and 52.6% female participants in the total cohort, and no significant differences in terms of the sex ratio were noted (*p* = 0.77). Although the body mass index (BMI) exhibited a trend toward higher values in the RDP group (27.6 ± 5.6) compared to the HALDP group (25.5 ± 5.8), this difference did not reach statistical significance (*p* = 0.08). Preoperative diabetes mellitus was present in 40.4% of the total cohort, with no significant disparities between groups (*p* = 0.55). Smoking status revealed a noteworthy finding: 35% of patients in the RDP group were smokers, compared to only 8.3% in the HALDP group, highlighting a significant difference (*p* = 0.03). Furthermore, preoperative albumin levels were comparable between the two groups, with a mean of 4.3 ± 0.4 in the total cohort (*p* = 0.32). The indications for surgery included a variety of conditions, such as carcinoma, neuroendocrine tumors, mucinous cystic neoplasms, and intraductal papillary mucinous neoplasms, with no significant differences in the distribution of these indications between groups (*p* = 0.19).

**Figure 1:**
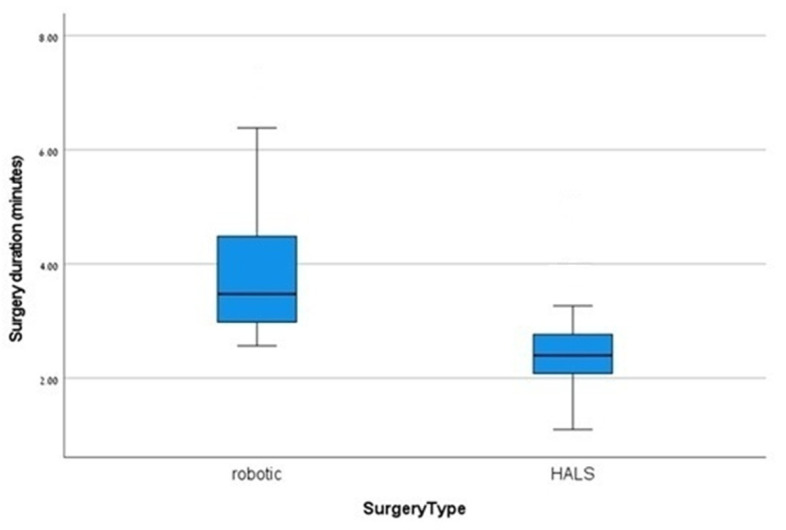
Operation time.

**Figure 2:**
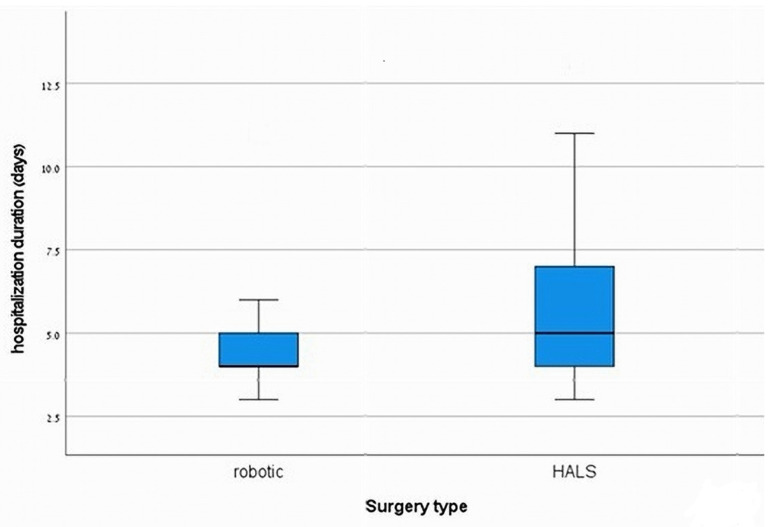
Length of hospital stay.

**Figure 3:**
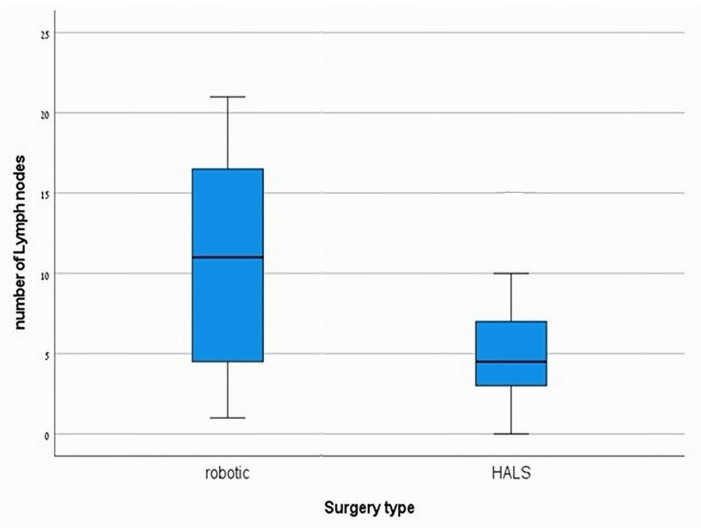
Number of harvested lymph nodes.

The perioperative characteristics are presented in Table 2. A significant finding was the extended operation time associated with RDP, averaging 3.9 ± 1.25 h compared to 2.5 ± 0.83 h for HALDP, with a *p*-value of <0.001 (Figure 1), indicating a considerable difference in surgical duration. Importantly, there were no conversions to open surgery in the RDP group, while 8.1% of patients in the HALDP group required conversion, although this difference was not statistically significant (*p* = 0.55). Estimated blood loss (EBL) was lower in the RDP cohort (160 ± 71.1 mL) compared to the HALDP group (260 ± 179.5 mL); however, this did not achieve statistical significance (*p* = 0.06). The length of stay (LOS) following surgery was significantly shorter for patients in the RDP group (4.7 ± 1.7 days) versus the HALDP group (5.8 ± 2.1 days) (*p* = 0.02) (Figure 2), suggesting improved recovery times with robotic assistance. Additionally, the number of harvested lymph nodes was significantly greater in the RDP group (11 ± 6.6) compared to the HALDP group (5.4 ± 4.4), with a *p*-value of 0.01 (Figure 3), indicating a potential advantage in oncological outcomes with robotic techniques.

Postoperative outcomes are detailed in Table 3. The incidence of clinically significant POPF (grades B and C) was observed in 35% of the RDP group and 16.2% of the HALDP group, although this difference did not reach statistical significance (*p* = 0.18). When considering all grades of POPF, rates remained similar between the surgical approaches (*p* = 0.51).

The overall complication rate was 47.4%, with 55% of the RDP group experiencing complications compared to 43.2% in the HALDP group; this difference was not statistically significant (*p* = 0.39). However, the analysis of 90-day postoperative morbidity, categorized using the Clavien–Dindo classification system, revealed a significant finding: 5% of the total cohort experienced a grade IIIa or higher morbidity, with none reported in the RDP group, while 8% of the HALDP group experienced similar complications (*p* = 0.04). This suggests a trend toward reduced rates of severe morbidities associated with robotic surgery. There was no 90-day mortality in either group. The readmission rates within 30 days postoperatively were comparable between the groups, with no significant differences (*p* = 0.29). Lastly, the follow-up duration was significantly longer for patients who underwent HALDP (44.3 ± 42.4 months) compared to those in the RDP group (8 ± 6.6 months), with a *p*-value of <0.001, indicating a marked difference in follow-up periods, which is attributed to the recent implementation of the robotic platform in our practice.

## 4. Discussion

Our study offers a unique comparison of RDP and HALDP in patients undergoing distal pancreatectomy. The results indicate that the RDP approach offers superiority on several levels while also validating the HALDP approach as a viable option with outcomes comparable to other surgical techniques, including robotic surgery.

The demographic and clinical characteristics of patients were largely similar between RDP and HALDP groups, with no significant differences in age, sex, BMI, diabetes rates, or preoperative albumin levels. However, a notable difference in smoking status was observed between the two groups, with 35% of patients in the RDP group being smokers, compared to only 8.3% in the HALDP group. Histological diagnoses were also similar between the two groups, although the RDP cohort showed a slight, non-significant increase in the proportion of more aggressive tumor types, such as pancreatic carcinomas and neuroendocrine tumors. This may be attributed to the growing use of robotic surgeries and the increasing application of advanced preoperative diagnostic techniques, such as magnetic resonance imaging and endoscopic ultrasound, which enable more accurate preoperative diagnosis and support a conservative approach for benign or less aggressive lesions [18].

A key finding of our study is the significantly longer operative time associated with RDP. We acknowledge this as a major drawback of the technique. The impact of this extends beyond the individual patient case and carries significant opportunity costs for the institution. The increased time required for an RDP procedure occupies valuable operating room resources, staff, and equipment, which can decrease overall surgical throughput and potentially delay access to care for other patients. This system-level economic and efficiency impact is a critical factor that institutions must weigh heavily when adopting and allocating robotic platforms. This finding is consistent with several studies highlighting longer operative times for RDP compared to other laparoscopic approaches [10,19,20,21,22]. The extended duration can be attributed to two main factors: (A) the time needed for instrument preparation and robotic system docking and (B) the learning curve associated with robotic procedures [23,24]. Existing evidence indicates that robotic surgery times at our institution will decrease as surgeons gain more experience with the robotic platform. Studies have shown that as surgeons become more proficient with the robotic system, the initially longer setup and operative times significantly improve. For instance, the average setup time decreased from 120 min for a single surgeon to just 8 min for a three-member team on their fourth setup. Furthermore, operative times decreased by an average of 26.6% after the second successive practice operation and by 39.0% after the third, making them comparable to those of conventional laparoscopic approaches [23].

The rate of conversion to open surgery did not significantly differ between the two surgical approaches, with three patients in the HALDP group requiring conversion compared to none in the RDP group. Similarly, while the estimated blood loss was not statistically significant between the two groups (*p* = 0.06), the RDP group showed a numerically lower median estimated blood loss. Several studies have shown that HALDP is associated with reduced intraoperative blood loss and fewer blood transfusions compared to open and pure laparoscopic surgery [6,7,25]. Furthermore, consistent with our findings, a study by Gamboa et al. demonstrated that the HALDP approach resulted in significantly lower blood loss than the open approach. In addition, there was higher blood loss compared to a combined group of minimally invasive procedures, including both pure laparoscopic and robotic approaches, which was not statistically significant [6]. Multiple studies and meta-analyses have consistently highlighted the robotic platform’s advantages in reducing blood loss and minimizing the need for conversion to open surgery, particularly when compared to both open and laparoscopic techniques [2,11,26,27]. Nonetheless, as our results indicate, the robotic approach’s superiority in reducing conversion rates and blood loss was not statistically significant when compared to the hand-assisted approach. This can be attributed to the unique benefits offered by both robotic and hand-assisted laparoscopy techniques, which reduce the likelihood of conversion to open surgery due to bleeding or technical complications. The hand-assisted laparoscopy technique provides the surgeon with tactile feedback and the ability to palpate the pancreatic tissue, which can facilitate complex retraction and quicker control of bleeding through digital pressure [6]. The robotic system’s enhanced dexterity, precision, and 3D visualization allow for more controlled and meticulous tissue dissection, especially around delicate structures such as blood vessels [28].

In terms of oncological outcomes, there was no statistically significant difference in margin-free (R0) resection between the two groups. However, it is worth noting that the RDP group achieved a 100% rate of R0 resections. Additionally, the number of harvested lymph nodes was significantly higher in the RDP group, approximately double that of the HALDP group. These results are consistent with several studies and meta-analyses that have suggested that RDP procedures may offer superior oncological outcomes, with higher R0 resection rates and a greater number of harvested lymph nodes compared to pure laparoscopic and open surgeries [26,29]. We do not attribute this to a difference in surgical intent, as our standardized technique—a radical antegrade pancreatosplenectomy—was uniformly applied to all patients regardless of preoperative diagnosis. This approach is standard in our practice due to the potential for occult malignancy even in presumed pre-cancerous lesions. Instead, we posit that this result demonstrates a key technological advantage of the RDP approach. The superior 3D visualization and enhanced dexterity of the robotic platform may allow for a more precise and meticulous dissection within this standardized oncologic field. This technological capability may be particularly impactful in adenocarcinoma cases, which were more frequent in our RDP cohort and can present with more prominent lymphadenopathy, thus allowing for a more thorough retrieval.

Furthermore, RDP has been associated with shorter hospital stays compared to both open and laparoscopic approaches [2,6,7,13], a finding confirmed in our study, where the RDP group had a significantly shorter length of hospital stay compared to the HALDP group. This indicates that the robotic approach may contribute to faster postoperative recovery, earlier discharge, and better resource utilization.

Although the overall complication rates were similar between the RDP and HALDP groups, the incidence of Clavien–Dindo grade IIIa or higher complications was significantly lower in the RDP group. This suggests that robotic surgery may have an advantage in reducing the incidence of severe postoperative complications. Further studies with larger sample sizes are needed to confirm this observation. The absence of 90-day mortality in both groups is consistent with the low mortality rates typically reported in the literature for distal pancreatectomies, regardless of the surgical approach [5,30].

Our findings regarding postoperative pancreatic fistulae (POPFs) align with those of other studies and meta-analyses, which have shown no significant differences in overall or clinically relevant (B/C) POPF rates between robotic and other surgical approaches [11,13,27,31]. A study by Romero et al. [7] comparing open, hand-assisted, and laparoscopic techniques for distal pancreatectomy found no difference in the incidence of clinically relevant (B/C) POPFs. However, a meta-analysis by Lyu et al. suggested that the robotic approach may have the lowest probability of both POPFs and clinically relevant (B/C) POPFs. Despite these mixed findings, some studies have reported no significant difference in POPF rates. Additionally, there are limited data on other factors that may influence POPFs, such as the pancreatic texture and closure techniques [26]. These findings indicate that the surgical approach itself may not be a major factor in the development of POPFs. Further research is needed to better understand the impact of robotic surgery on the incidence of POPFs.

To further improve outcomes in distal pancreatectomy, future research should also explore novel intraoperative assessment modalities that could provide real-time anatomical or physiological data on the pancreatic remnant. Additionally, the role of preoperative hematological inflammatory markers, such as the neutrophil-to-lymphocyte ratio, warrants investigation for better patient risk stratification. These avenues represent important future directions for optimizing patient care, regardless of the surgical platform used.

Our study’s limitations include its retrospective, single-center design and the comparison of a contemporary robotic cohort with a historical HALDP control group. This sequential design introduces potential temporal bias from unmeasured confounders, such as the evolution of our ERAS protocol and other aspects of perioperative care. Furthermore, our analysis is limited by the lack of patient-reported outcome measures (PROMs), such as data on return to normal activities, which are needed to more comprehensively assess the full clinical benefit and recovery experience. Finally, the shorter follow-up for the RDP group prevents long-term analysis. These factors highlight the need for larger, multicenter prospective studies that incorporate PROMs to validate our findings.

## 5. Conclusions

Our comparative study of RDP and HALDP reveals a nuanced trade-off between these two minimally invasive techniques. The clear advantage of HALDP was a significantly shorter operative time. In contrast, RDP was associated with certain favorable outcomes, including a significantly shorter hospital stay and a higher number of harvested lymph nodes. Most notably, the RDP group experienced a significantly lower rate of severe postoperative complications (Clavien–Dindo grade ≥ IIIa). Other key metrics, including overall complication rates and R0 resection rates, were comparable between the groups. These results suggest that while RDP has the considerable disadvantage of longer operative times, it may offer distinct advantages for patient recovery. HALDP remains a safe and efficient alternative. Further large-scale, prospective studies are essential to validate these findings and to better define the optimal role of each approach in the management of distal pancreatic lesions.

## Figures and Tables

**Table 1:** Patient characteristics.

Variable	Total (N = 57)	RDP (N = 20)	HALDP (N = 37)	*p*
Age (years)	64.1 ± 13.7	65.2 ± 9.9	63.5 ± 15.5	0.65
Sex				0.77
M	27 (47.4%)	10 (50%)	17 (45.9%)	
F	30 (52.6%)	10 (50%)	20 (54.1%)	
Body mass index	26.7 ± 5.7	27.6 ± 5.6	25.5 ± 5.8	0.08
Preoperative diabetes mellitus				0.55
Yes	23 (40.4%)	7(35%)	16 (43.2%)	
No	34 (59.6%)	13(65%)	21 (56.8%)	
Smoking status				0.03
Yes	10 (17.9%)	7 (35%)	3 (8.3%)	
No	46 (82.1%)	13 (65%)	33 (91.7%)	
Preoperative albumin level	4.3 ± 0.4	4.4 ± 0.52	4.3 ± 0.33	0.32
Indication for surgery				0.19
Ca	16 (28.1%)	9 (45%)	7 (18.9%)	
NET	13 (22.8%)	5 (25%)	8 (21.6%)	
MCN	9 (15.8%)	1 (5%)	8 (21.6%)	
IPMN	5 (8.8)	1 (5%)	4 (10.8%)	
Other	14 (24.6%)	4 (20%)	10 (27%)	

RDP, robotic-assisted distal pancreatectomy; HALDP, hand-assisted laparoscopic distal pancreatectomy; Ca, carcinoma; NET, neuroendocrine tumor; MCN, mucinous cystic neoplasm; IPMN, intraductal papillary mucinous neoplasm.

**Table 2:** Perioperative characteristics.

Variable	RDP (N = 20)	HALDP (N = 37)	*p*
Operation time (h)	3.9 ± 1.25	2.5 ± 0.83	<0.001
Conversion to open			0.55
Yes	0 (0%)	3 (8.1%)	
EBL (mL)	160 ± 71.1	260 ± 179.5	0.06
Perioperative blood transfusion			>0.99
Yes	1 (5%)	1 (2.7%)	
No	19 (95%)	36 (97.3%)	
LOS	4.7 ± 1.7	5.8 ± 2.1	0.02
R0 resection %	20 (100%)	35 (94.6%)	0.54
Number of harvested lymph nodes	11 ± 6.6	5.4 ± 4.4	0.01

RDP, robotic-assisted distal pancreatectomy; HALDP, hand-assisted laparoscopic distal pancreatectomy; LOS, length of stay; EBL, estimated blood loss.

**Table 3:** Postoperative outcomes.

Variable	Total (N = 57)	RDP (N = 20)	HALDP (N= 37)	*p*
Clinically significant POPF (grade B/C)				0.18
Yes	13 (22.8%)	7 (35%)	6 (16.2%)	
No	44 (77.2%)	13 (65%)	31 (83.8%)	
POPF (all grades)				0.51
Yes	28 (49.1%)	11 (55%)	17 (45.9%)	
No	29 (50.9%)	9 (45%)	20 (54.1%)	
Complications, yes, n (%)	27 (47.4%)	11 (55%)	16 (43.2%)	0.39
90-day postoperative morbidity (Clavien–Dindo Grade) ≥ IIIa	3 (5%)	0 (0%)	3 (8%)	0.04
Readmission in 30 days, yes, n (%)	10 (17.5%)	5 (25%)	5 (13.5%)	0.29
Follow-up (month)	30.6 ± 37.9	8 ± 6.6	44.3 ± 42.4	<0.001

RDP, robotic-assisted distal pancreatectomy; HALDP, hand-assisted laparoscopic distal pancreatectomy; POPF, postoperative pancreatic fistula.

## Data Availability

The data presented in this study are available on request from the corresponding author due to legal reasons.
